# Guggulsterone Targets Smokeless Tobacco Induced PI3K/Akt Pathway in Head and Neck Cancer Cells

**DOI:** 10.1371/journal.pone.0014728

**Published:** 2011-02-24

**Authors:** Muzafar A. Macha, Ajay Matta, Shyam Singh Chauhan, K. W. Michael Siu, Ranju Ralhan

**Affiliations:** 1 Department of Biochemistry, All India Institute of Medical Sciences, New Delhi, India; 2 Department of Chemistry and Center for Research In Mass Spectrometry, York University, Toronto, Ontario, Canada; 3 Alex and Simona Shnaider Laboratory of Molecular Oncology, Mount Sinai Hospital, Toronto, Ontario, Canada; 4 Joseph and Mildred Sonshine Family Centre for Head & Neck Diseases, Mount Sinai Hospital, Toronto, Ontario, Canada; 5 Department of Otolaryngology - Head and Neck Surgery, Mount Sinai Hospital, Toronto, Ontario, Canada; 6 Department of Pathology and Laboratory Medicine, Mount Sinai Hospital, Joseph & Wolf Lebovic Health Complex, Toronto, Ontario, Canada; 7 Department of Otolaryngology - Head and Neck Surgery, University of Toronto, Toronto, Ontario, Canada; University of Dayton, United States of America

## Abstract

**Background:**

Epidemiological association of head and neck cancer with smokeless tobacco (ST) emphasizes the need to unravel the molecular mechanisms implicated in cancer development, and identify pharmacologically safe agents for early intervention and prevention of disease recurrence. Guggulsterone (GS), a biosafe nutraceutical, inhibits the PI3K/Akt pathway that plays a critical role in HNSCC development. However, the potential of GS to suppress ST and nicotine (major component of ST) induced HNSCC remains unexplored. We hypothesized GS can abrogate the effects of ST and nicotine on apoptosis in HNSCC cells, in part by activation of PI3K/Akt pathway and its downstream targets, Bax and Bad.

**Methods and Results:**

Our results showed ST and nicotine treatment resulted in activation of PI3K, PDK1, Akt, and its downstream proteins - Raf, GSK3β and pS6 while GS induced a time dependent decrease in activation of PI3K/Akt pathway. ST and nicotine treatment also resulted in induction of Bad and Bax phosphorylation, increased the association of Bad with 14-3-3ζresulting in its sequestration in the cytoplasm of head and neck cancer cells, thus blocking its pro-apoptotic function. Notably, GS pre-treatment inhibited ST/nicotine induced activation of PI3K/Akt pathway, and inhibited the Akt mediated phosphorylation of Bax and Bad.

**Conclusions:**

In conclusion, GS treatment not only inhibited proliferation, but also induced apoptosis by abrogating the effects of ST / nicotine on PI3K/Akt pathway in head and neck cancer cells. These findings provide a rationale for designing future studies to evaluate the chemopreventive potential of GS in ST / nicotine associated head and neck cancer.

## Introduction

Head and neck squamous cell carcinoma (HNSCC) remains a significant cause of morbidity and mortality worldwide with five year survival rates of about 50%, marred by frequent recurrence or formation of second primary tumors (10–25% of cases)[1], [2]. On a global scale, the use of tobacco products is the major risk factor, with smokeless tobacco (ST) consumption being linked to the high incidence of HNSCC [3]-[5]. ST is used in multiple forms namely naswar, gutkha, khaini (a mixture of ST with lime) and has or with betel quid, been classified as a human carcinogen by International Agency of Research in Cancer (IARC) [6], [7]. The association of smoking with HNSCC is well known, but the link between ST use and head and neck cancer is emerging. In a recent report, Stepanov *et al.* [8] identified 23 polycyclic aromatic hydrocarbons (PAH) in ST in addition to nitrosamines and nicotine as reported in earlier studies [7]. Nicotine enhances proliferation, accelerates tumor growth and inhibits apoptosis in certain types of human cancer cell lines and induces angiogenesis in vivo by sustained activation of the mitogenic pathways [9]-[11]. Nicotine has been reported to inhibit apoptosis induced by opioids and genotoxic stress induced by etoposide, cisplatin, or UV irradiation in lung cancer cells [12], [13]. In addition, nicotine activates PI3K/Akt pathway and inhibits the pro-apoptotic functions of Bax and Bad through phosphorylation [14], [15]. These findings prompted us to investigate whether ST (khaini) induces PI3K/Akt pathway activation in head and neck cancer cells.

Further, identification of pharmacologically safe chemopreventive agents that can suppress ST/nicotine induced PI3K/Akt pathway in HNSCC is likely to have the potential to prevent ST induced head and neck carcinogenesis. Guggulsterone (GS), (4,17(20)-pregnadien- 3,16-dione), a constituent of Indian Ayurvedic medicinal plant *Commiphora mukul* is a biosafe nutraceutical with anti-neoplastic properties [16]-[18]. GS has been reported to induce apoptosis, suppress proliferation, invasion, angiogenesis, metastasis and reverse drug resistance, making it a potential complementary anti-cancer agent [19]-[24]. GS binds to farnesoid X receptor and has been shown to modulate expression of proteins involved in apoptosis (IAP1, XIAP, Bfl-1/A1, Bcl-2, cFLIP, survivin), cell proliferation (cyclin D1, c-Myc), angiogenesis and metastasis (MMP-9, COX-2, VEGF) [25]. This sterol exerts its biologic effects by regulation of transcription factors – nuclear factor kappa B (NFκB), STAT-3 and C/EBP alpha, and steroid receptors - glucocorticoid receptors and androgen receptor. Our group and others recently demonstrated anticancer activity of GS in HNSCC and myeloma cell lines by inhibition of NFκB and STAT3; subsequent studies reported similar effects in other cancer cell lines and in animal models of colon and skin cancer [19], [24], [26].

In this study, we investigated the activation of PI3K/Akt pathway in head and neck cancer cells on treatment with ST/nicotine and its inactivation by GS. Further we challenged activation of Akt and its downstream targets (pS6, GSK3β and Raf), by ST/nicotine in head and neck cancer cells (SCC4) by pre-treatment with GS.

## Results

### Effect of ST and nicotine activate the PI3K/Akt pathway in head and neck cancer cells

**Figure 1:**
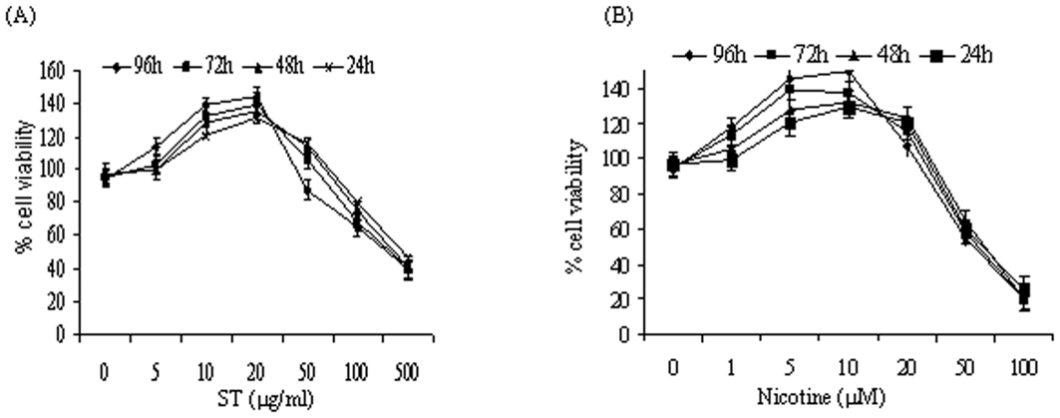
Dose and Time dependent kinetics of ST and nicotine treatment on SCC4 cells using MTT assay. Cells were treated with (A) ST (1-500 µg/ml ), (B) nicotine (1-100 µM) for 24–96 h. Figure depicts average of three experiments done independently.

**Figure 2:**
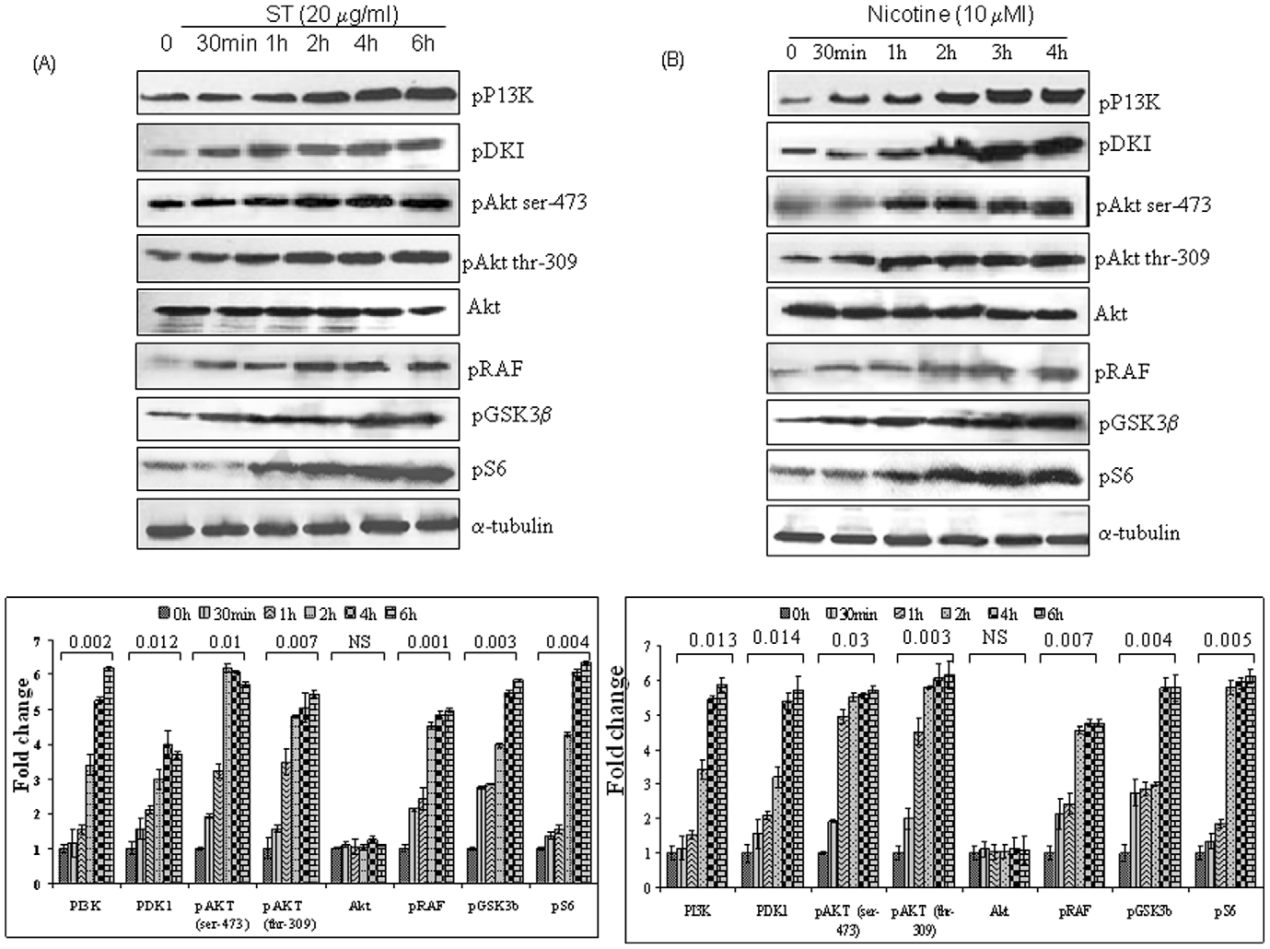
ST and nicotine - induced Akt pathway activation. SCC4 cells (2–3×10^6^) were treated with (**A**) ST (20 µg/ml) or (**B**) nicotine (10 µM), for the indicated time intervals and whole-cell extracts were prepared. Whole-cell extracts (60 µg protein) were resolved on 10% SDS-PAGE, electrotransferred to a PVDF membrane and non-specific binding was blocked with 5% non-fat milk overnight. Protein expression was determined by probing with phospho-specific antibodies for pAkt (thr-309), pAkt (ser-473). pGSKβ3, pRaf, pPDK1 and Akt using enhanced chemiluminescence method. Western blotting for α-tubulin was done to show equal protein loading.

The dose and time kinetics of ST and nicotine (addictive component of tobacco) treatment in head and neck cancer cells (SCC4 and HSC2), were determined by MTT assay (Figure 1A and 1B). Similar kinetics was observed in both cell lines and the results for SCC4 cells are shown in Figure 1. Exposure to ST and nicotine increased cell proliferation in both the head and neck cancer cell lines in a dose dependant manner with an optimal dose of 20 µg/ml for ST and 10 µM for nicotine. These optimal doses have been used in all the subsequent experiments. To investigate the effect of ST or nicotine on the Akt pathway in HNSCC cells, both SCC4 and HSC2 cells were treated with 20 µg/ml of ST or with nicotine (10 µM) for different time intervals or kept untreated, and Akt activation (Akt phosphorylation at Ser-473 and Ser-309) was determined in the protein extracts by western blotting. Both ST and nicotine rapidly increased Akt phosphorylation without affecting the total Akt levels in both HSC2 and SCC4 cells (Figure 2A and 2B respectively). These studies demonstrate that ST and nicotine activate Akt at doses that could be physiologically relevant. Further, our western blot analysis showed both ST and nicotine induced a time-dependent increase in phosphorylation of both the upstream kinases, PI3K and PDK1, as well as the downstream targets, Raf, GSK3β, and pS6 in SCC4 cells (Figure 2A and 2B), that reflected the rapid onset of increased Akt phosphorylation. Similar findings were observed in both the cell lines (SCC4 and HSC2) therefore, data for only SCC4 cells have been shown here.

### Effect of guggulsterone on ST and nicotine induced activation of PI3K/Akt pathway

**Figure 3:**
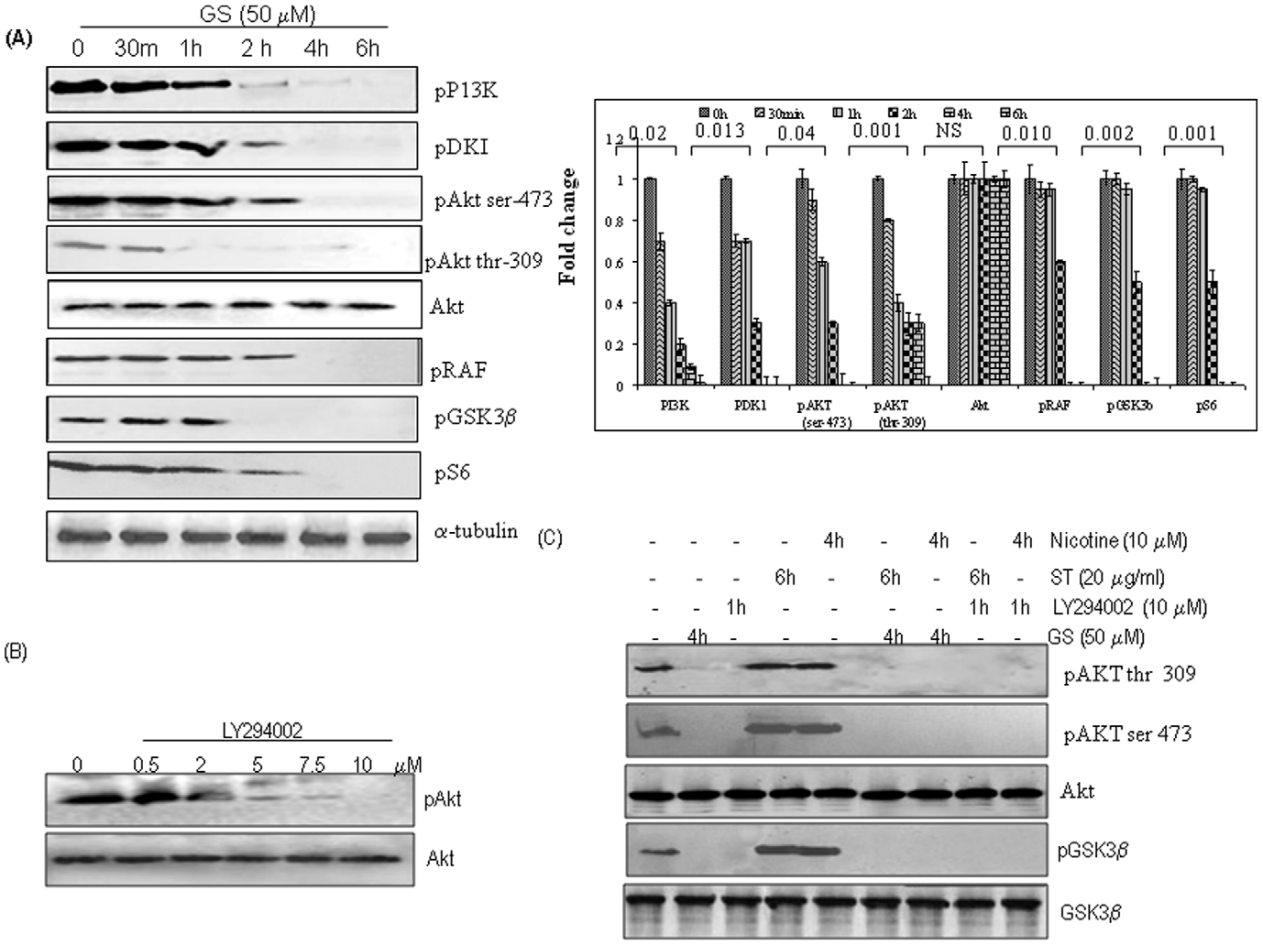
Guggulsterone inhibits constitutive and ST or nicotine induced Akt pathway activation. SCC4 cells (2–3×10^6^) were treated with (**A**) GS (50 µM) for the indicated time intervals and/ or (**B**) with LY294002 at different concentrations for 1 h. (**C**) SCC4 cells (2–3×10^6^) were pre-treated with 50 µM GS for 4 h or with LY294002 (10 µM) for 1 h and stimulated with ST (20 µg/ml) for 6 h, or with nicotine (10 µM) for 4 h. Sixty micrograms of proteins from whole-cell extracts were resolved on 10% SDS-PAGE, electrotransferred to a PVDF membrane followed by blocking with 5% non-fat milk overnight. Protein expression was determined by probing with specific antibodies using enhanced chemiluminescence method.

Both ST and nicotine induced stimulation of Akt pathway, therefore, we investigated the effect of GS on ST and nicotine induced activation of Akt pathway. SCC4 cells were treated with GS (50 µM) for different time intervals and their protein extracts were tested for Akt activation. As shown in Figure 3A, GS inhibited the activation of Akt, upstream kinases PI3K, PDK1, as well as the downstream proteins, Raf, GSK3β, and pS6. The dose standardization of PI3K/Akt inhibitor, LY294002, was carried out by treating the SCC4 cells with different concentrations of the inhibitor (0.5–10 µM) for 1 h. As shown in the Figure 3B, LY294002 (10 µM) completely inhibited the expression of activated Akt; while no effect was observed on the total Akt expression levels. SCC4 cells were pre-treated with GS for 4 h or LY294002 (10 µM) for 60 min, followed by ST (20 µg/ml) for 6 h or nicotine (10 µM) for 4 h. The results indicate that GS as well as LY294002 blocked ST and nicotine-induced Akt pathway activation (Figure 3C), though no effect was observed on the expression of total Akt and GSK3β at these time points.

### Guggulsterone and PI3K-specific inhibitor LY294002 block ST and nicotine induced Bad and Bax phosphorylation

**Figure 4:**
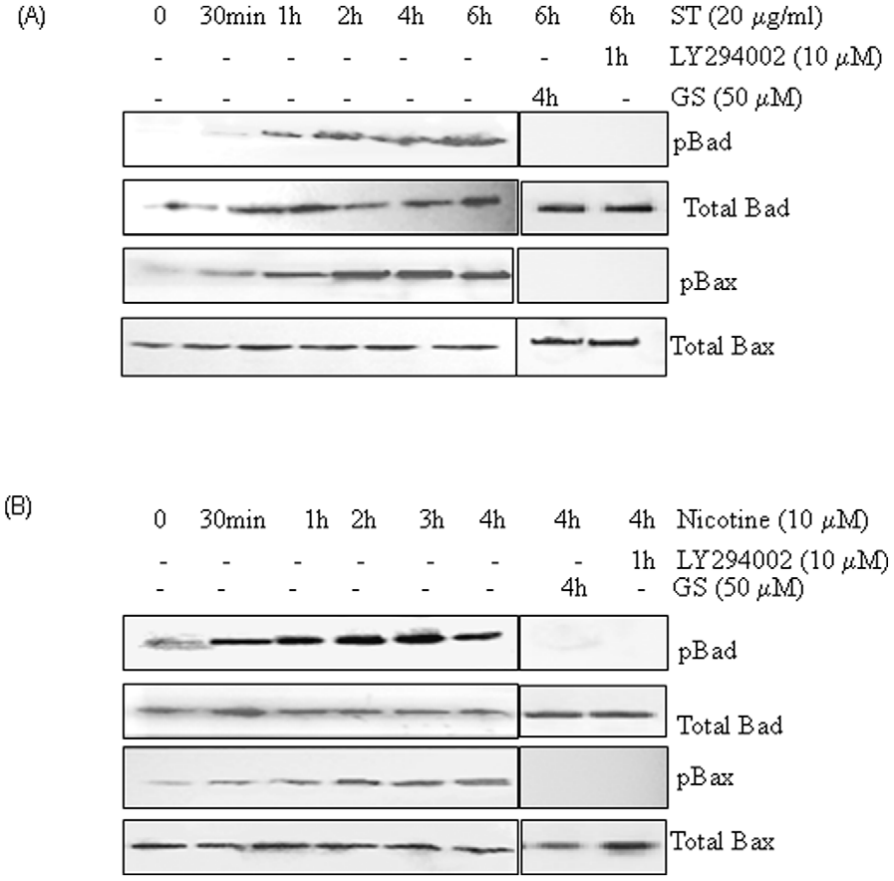
Guggulsterone and PI3K-specific inhibitor LY294002 block ST and nicotine induced Bad and Bax phosphorylation. SCC4 cells (2–3×10^6^) were treated with (A) ST (20 µg/ml) or (B) Nicotine (10 µM) for the indicated time intervals. SCC4 cells were pre-treated with LY294002 (10 µM) for 60 min, or 50 µM GS for 4 h, followed by ST (20 µg/ml) for 4 h, or with nicotine (10 µM) for 4 h, and whole-cell extracts were prepared. Sixty microgram proteins from whole-cell extracts were resolved on 10% SDS-PAGE, electrotransferred to a PVDF membrane followed by blocking with 5% non-fat milk overnight. Protein expression was determined using enhanced chemiluminescence method and probed by antibodies against pBad and pBax. The blots were stripped and reprobed for Bax and Bad proteins.

Both ST and nicotine potently stimulated serine phosphorylation of Bax and Bad in a time dependent manner (Figure 4A and 4B) resulting in abrogation of their pro-apoptotic function. To demonstrate a functional role of Akt as the physiological ST and nicotine activated Bax and Bad kinase, LY294002, a PI3K specific inhibitor that can block the PI3K/Akt signaling pathway, was used. SCC4 cells were pre-treated with LY294002 (10 µM) for 60 min or 50 µM GS for 4 h followed by ST (20 µg/ml) for 6 h or with nicotine (10 µM) for 4 h. GS as well as LY294002 blocked ST and nicotine induced Bax and Bad phosphorylation (Figure 4A and 4B). No effect on expression of total Bad and Bax was observed at these time points. These findings suggest that inhibition of ST and nicotine induced Bax and Bad phosphorylation by GS may restore the pro-apoptotic function of these molecules.

### Akt is co-localized with Bax in cytoplasm

**Figure 5:**
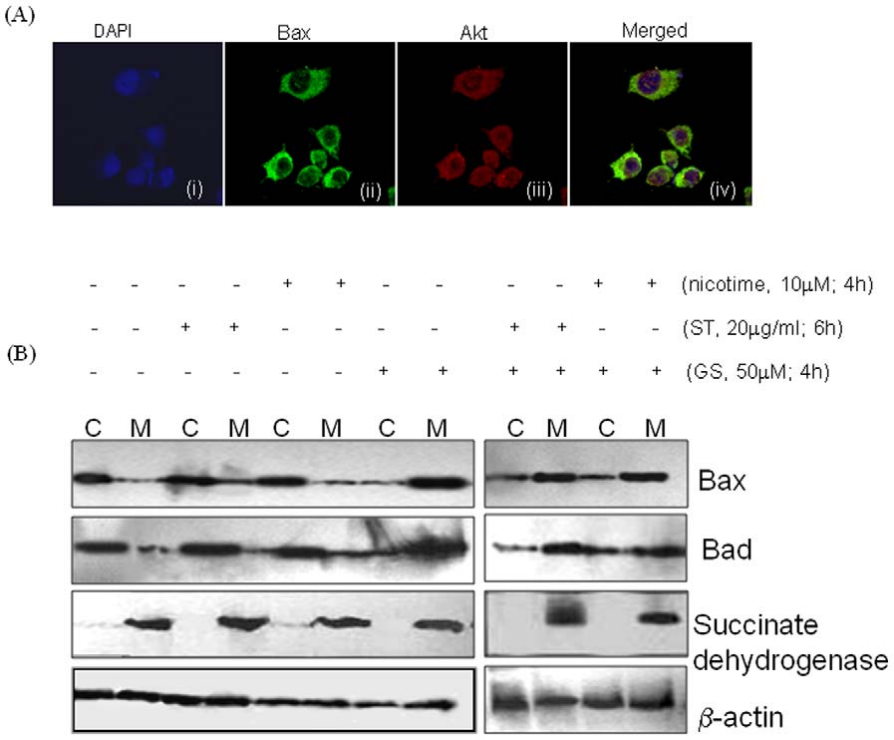
(A) AKT is co-localized with Bax in cytoplasm. SCC4 cells (5×10^3^) were plated on coverslips and incubated with a mouse antibody against human Bax and a rabbit antibody against human AKT antibodies. Alexa flour®594 -conjugated anti-rabbit (red) and Fluorescein isothiocyanate-conjugated (green) anti-mouse secondary antibodies were used to visualize Akt (red) and Bax (green) localization patterns using a fluorescent microscope. Panel (i) DAPI stained nuclei in blue color; (ii) cytoplasmic expression of Bax; (iii) cytoplasmic expression of Akt protein; and (iv) merged photomicrograph (ii) and (iii) showing co-localization of Bax and Akt. **(B)** Phosphorylation of Bax at (Ser-184) and Bad (Ser-136) results in retention of Bax and Bad in cytosol. SCC4 cells were kept untreated or treated with 50 µM GS for 4 h, ST (20 µg/ml) for 6 h, 10 µM nicotine for 4 h. SCC4 cells were pre-treated with 50 µM GS for 4 h, followed by ST for 6 h or with nicotine for 4 h. Cytoplasmic (C) and Mitochondrial (M) extracts were prepared as described in materials and methods and separated on 10% SDS-PAGE. Proteins were then electro-transferred on PVDF membrane followed by blocking with 5% non-fat milk overnight. Blots were incubated with specific antibodies against Bax and Bad. Protein expression was determined using enhanced chemiluminescence method. Purity of the subcellular fractions obtained was determined using western blot for mitochondrial protein, succinate dehydrogenase. β-actin was used as a loading control.

To assess a potential direct role for Akt as a physiological Bax kinase, sub-cellular distribution of Akt and Bax were assessed by immunofluorescence staining. Bax was primarily co-localized with Akt in the cytoplasm of SCC4 cells (Figure 5A) suggesting that both ST and nicotine activated Akt has the potential to directly phosphorylate and inactivate Bax in SCC4 cells.

### Phosphorylation of Bax and Bad results in retention of these proteins in cytosol and failure to target mitochondria

Our results demonstrated that ST and nicotine can induce Bax (Ser-184) and Bad (ser-136) phosphorylation, and phosphorylation at these sites resulted in inactivation of the pro-apoptotic function of Bax and Bad. It is well known that the pro-apoptotic activity of Bax and Bad is dependent on its cytosolic or mitochondrial localization. To test whether phosphorylation of Bax and Bad effects its sub-cellular localization, SCC4 cells were kept untreated or treated with GS (50 µM) for 4 h, ST (20 µg/ml) for 6 h and nicotine (10 µM) for 4 h. Sub-cellular fractionations were performed to isolate mitochondria and cytosol as described under material and methods. In untreated SCC4 cells, majority of Bax and Bad was found in the cytosol, and only a small amount of these proteins, was localized in the mitochondria (Figure 5B). In GS treated cells majority of these proteins were observed in the mitochondrial fraction. In contrast, in both ST and nicotine treated cells, Bax and Bad were predominantly observed in the cytosol (Figure 5B).

Notably, pre-treatment of SCC4 cells with GS (50 µM) for 4 h, followed by ST (20 µg/ml) treatment for 6 h or nicotine (10 µM) for 4 h, showed a shift in localization of Bax and Bad and majority of these proteins were observed in the mitochondrial fraction (Figure 5B). To confirm the purity of the subcellular fractions obtained, succinate dehydrogenase, a protein exclusive to the mitochondria, was assessed by western blotting. Succinate dehydrogenase, was detected only in the mitochondrial fraction and not in the cytosol (Figure 5B), confirming the purity of the mitochondrial and cytosolic fractions. β-actin was used as the control to ensure equal protein loading in all the lanes.

### ST and nicotine induced Bad phosphorylation enhances interaction with 14-3-3ζ

**Figure 6:**
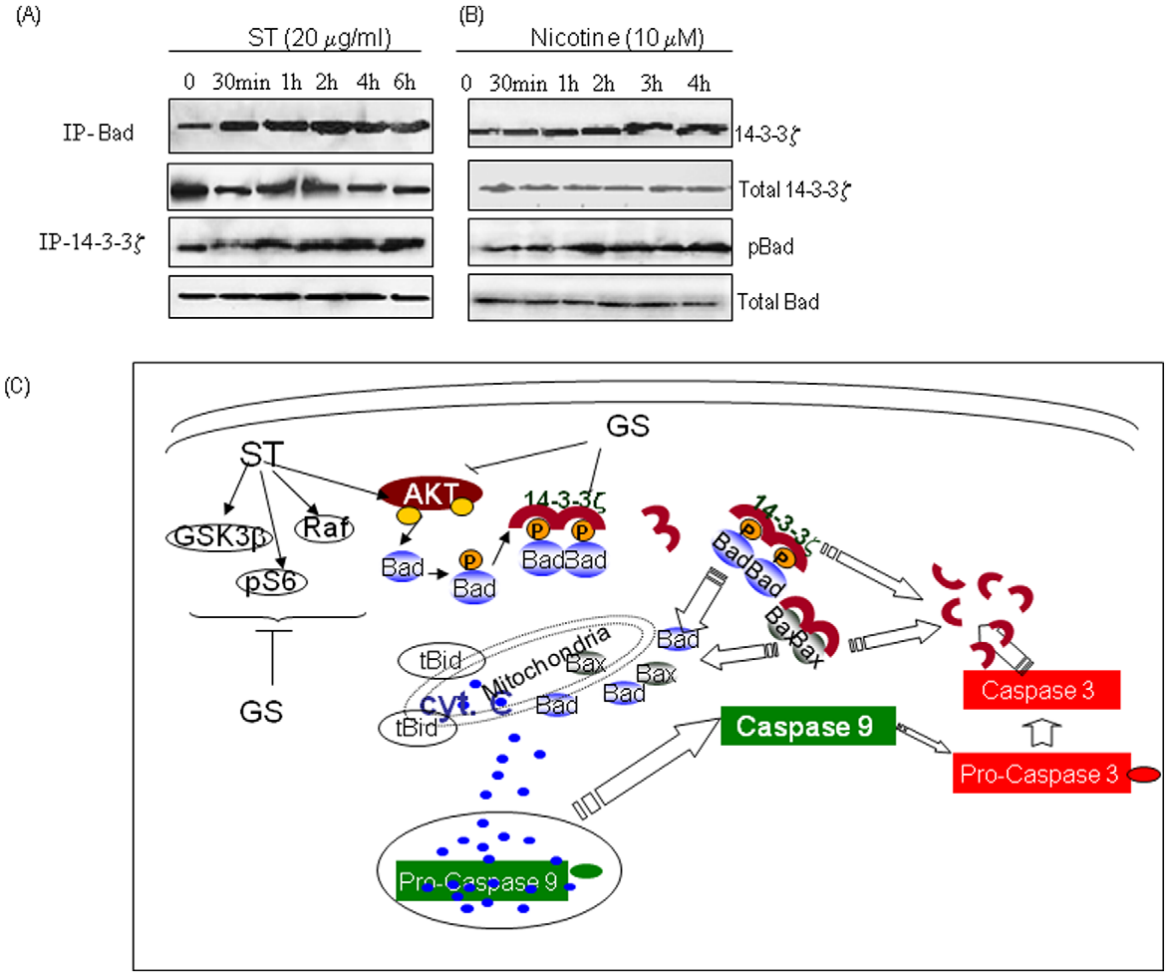
ST and nicotine induces Bad phosphorylation enhances interaction with 14-3-3ζ. SCC4 were treated with (**A**) ST (20 µg/ml) or (**B**) with nicotine (10 µM) for different time intervals and immunoprecipitation assays were carried out using whole cell lysates and analyzed by western blotting as described in Materials and Methods. 14-3-3ζ was immunoprecipitated using specific antibody and the bound pBad protein co-precipitated with 14-3-3ζ was determined by western blot analysis. Reverse immunoprecipitation assays were carried out in which pBad was immunoprecipitated, followed by western blotting analysis of 14-3-3ζ using a specific antibody against this protein. (**C**) Figure shows the hypothetical model for inhibition of ST / nicotine induced activation of PI3K/Akt pathway by GS. Our results showed treatment with ST and /or nicotine activates Akt (phosphorylated at Ser-473 and Ser-309) resulting in phosphorylation of its downstream targets Raf, GSK3β, and pS6, whereas GS treatment inhibits activation of Akt pathway. Interestingly, pre-treatment of head and neck cancer cells with GS inhibits activation of Akt and its downstream targets (Raf, GSK3β, and pS6) on exposure to ST / nicotine. GS pre-treatment also releases Bad from inhibitory action of 14-3-3ζ, thereby activating intrinsic pathway of apoptosis. Thus, our results demonstrated GS as a potential therapeutic agent for ST-induced head and neck carcinogenesis.

Bad phosphorylation (ser-136) promotes its translocation from mitochondria into cytosol and interaction with the scaffold protein 14-3-3. To assess whether ST or nicotine affect Bad association, SCC4 cells were treated with ST (20 µg/ml) or nicotine (10 µM) for different time intervals. Co-immunoprecipitation of 14-3-3ζ and Bad showed a time dependent increase in bound pBad in the immunocomplexes (IP) of 14-3-3ζ indicating association between 14-3-3ζ and pBad (Figure 6A and 6B), on treatment with ST and nicotine respectively. Similar results were observed in reverse immunoprecipitation assays, western blotting was done for 14-3-3ζ in immunocomplexes obtained using pBad specific antibody, confirming the interaction of 14-3-3ζ with pBad (Figure 6A and 6B). These results suggest ST and nicotine induced Bad phosphorylation resulted in sequestering Bad in the cytoplasm, functionally blocking its pro-apoptotic function.

## Discussion

Tobacco in various forms is one of the main etiological factors for development and progression of HNSCC. Aberrant expression of genes involved in cell growth, survival, angiogenesis, invasion and metastasis in ST associated head and neck carcinogenesis has been reported by our group and others [27]-[31]. In this study, we demonstrated activation of Akt in HNSCC cells treated with ST/nicotine, evidenced by sustained phosphorylation of Akt at serine 473 and threonine 308, as well as its downstream substrates (pS6, GSK3β, pRaf), in a time-dependent manner. Both ST and nicotine also induced the phosphorylation of the upstream kinase PDK1 (Ser241), and p85 subunit of another upstream kinase, PI3K in SCC4 cells. These observations are supported by the earlier reports showing activation of PI3K/Akt in cultured cortical neurons, normal human bronchial and small airway epithelial cells in response to nicotine treatment [11], [32]-[34]. Further, we showed both ST and nicotine activated Akt, induced Bad (Ser136) and Bax phosphorylation (Ser184) resulting in cytoplasmic retention of these proteins. However, treatment with LY294002, a PI3K/Akt inhibitor, potently inhibited ST and nicotine induced phosphorylation of Bad (Ser-136) and Bax (Ser-184) resulting in mitochondrial relocalization of these proteins, further strengthening our observation that ST and nicotine induced phosphorylation of Bad (Ser-136) and Bax (Ser-184) by activation of PI3K/Akt. Interestingly, both ST and nicotine induced phosphorylation of Bad (Ser-136) in SCC4 cells increased its association with 14-3-3ζ as revealed by co-IP assays. This results in sequestration of Bad in the cytoplasm, thus inhibiting the intrinsic pathway of apoptosis. Earlier reports have shown phosphorylation on either Ser112 or Ser136 facilitates formation of a complex between Bad and 14-3-3s in the cytosol, blocking their interaction with Bcl2/Bcl-xl in the mitochondria [35], [36].

Recently, we demonstrated overexpression of PI Synthase, PI3-K and cyclin D1 in ST treated cells from oral lesions and oral cancer [37]. Further, analyzing clinical specimens from oral leukoplakic lesions without dysplasia, with dysplasia and oral squamous cell carcinomas (OSCCs), we provided the first evidence of increased expression of PI Synthase in early stages of oral precancer and cancer and its correlation with tumor dedifferentiation and tobacco consumption [37]. Here in we extended these findings to demonstrate GS could inhibit the induction of PI3K/Akt by ST and nicotine.

Thus, inhibiting the induction of Akt activity by ST tobacco components might be a valuable approach to mitigate the effects of tobacco in consumers at risk for the development of HNSCC, and/or in HNSCC patients who continue to smoke or use ST products. In this respect, we observed that GS suppressed the phosphorylation of Akt (Ser473 and Thr308), PDK1 (Ser241) and p85 subunit of PI3K, leading to decreased phosphorylation of GSK3β, pRaf, pS6, the downstream targets of Akt. Notably, our study revealed that GS not only inhibited the constitutive PI3K/Akt pathway, but its pre-treatment abrogated both ST and nicotine induced activation of PI3K/Akt pathway. ST and nicotine abrogated the pro-apoptotic effects of Bax/Bad by phosphorylation and sequestering in cytoplasm, however GS pre-treatment inhibited the effect of ST and nicotine, targeting Bax and Bad to the mitochondria, inducing apoptosis. However, the effect of GS is not specific and limited to head and neck cancer cells only. We and others have shown the anti-cancer effects of GS in a variety of cancer cell types namely, head and neck; leukemia, multiple myeloma, lung, melanoma, ovarian, gut derived adenocarcinoma, colorectal, colon, skin, prostate, esophagus, and breast [19], [21]-[24], [38]-[43].

In conclusion, our study demonstrated that both ST and nicotine promote survival of human head and neck cancer cells SCC4, by inactivating the pro-apoptotic functions of Bax and Bad via its phosphorylation and sequestering these proteins in cytoplasm. GS not only inhibits constitutively active PI3K/Akt pathway, but also inhibits ST and nicotine induced activation of this pathway and promotes apoptosis signals in these cells (Figure 6c). Hence, GS may be explored as a chemopreventive agent for ST induced head and neck carcinogenesis.

## Materials and Methods

### Antibodies and Reagents

z-Guggulsterone (GS), 3-(4,5-dimethylthiazol-2-yl)-2,5-diphenyltetrazolium bromide (MTT), propidium iodide (PI) were purchased from Sigma-Aldrich (St. Louis, MO). Mouse monoclonal antibody against Bax (sc-7480); rabbit polyclonal antibody against 14-3-3ζ (sc-1019), pPI3K p85 antibody (Tyr-508, sc-12929R) were purchased from Santa Cruz Biotechnology, Santa Cruz, CA. Rabbit monoclonal antibody against phospho-Akt (Ser 473, cat-9271), phospho-Akt (Thr 308, cat-9275), Akt (cat-9272), phospho-GSK-3β (Ser 9, cat-9336), phospho-Raf (Ser 259, cat-9421), phospho-PDK1 (Ser 241, cat-3061), PI3-kinase inhibitor LY294002 (cat-9901) were all purchased from Cell Signaling Technology. For co-localization studies, goat anti-rabbit Alexa Fluor® 594 (Cat no A-11012) was purchased from Invitrogen (Carlsbad, CA). Goat anti-mouse FITC (Cat no. F2012) was purchased from Sigma (St. Louis, MO). Protein A-sepharose beads were obtained from GE Healthcare Biosciences, (Uppsala, Sweden).

### Cell culture

Human head and neck squamous carcinoma cell lines, SCC4 was obtained from American Type Culture Collection (ATCC) and HSC2 (JCRB0622) was obtained from Health Science Research Resources Bank (HSRRB), Japan [44], [45]. Both these head and neck cancer cell lines are known to harbor mutant p53. SCC4 cells have a point mutation at codon 51 CCC to TCC and HSC2 cells have a mutation in intron 6 [44], [45]. HSC-2 cells harbor mutated PIK3CA (H1047R) [46]. Both head and neck cancer cell lines (SCC4 and HSC2) were grown in monolayer cultures in Dulbecco's modified eagle medium (DMEM) (Sigma, St. Louis, MO) supplemented with 10% Fetal bovine serum (FBS) (Sigma), 1 mM L-glutamine, 1X sodium pyruvate, 1X vitamins, 1 mM minimum essential medium (MEM), 100 µg/ml streptomycin and 100 U /ml penicillin in a humidified incubator (5% carbon-dioxide, 95% air) at 37 °C as described earlier [47].

### Preparation of Smokeless Tobacco Extract (ST)

Commonly used brand of cured tobacco (Khaini) was purchased from the local market. 25 g of tobacco was finely powdered and homogenized in 225 ml of distilled water. Mixture was stirred on a magnetic stirrer for two hours and then allowed to stand for 24 hrs at 37 °C. Thereafter, supernatant was collected after centrifugation at 5000 g for 20 minutes. The extract was sterilized by passing it through a 0.22 µm filter and stored at 4 °C till use. The final concentration of tobacco in aqueous extract was estimated to be 2.7 g% [48].

### Cytotoxicity Assay

Head and neck cancer cells (5×10^3^/well) were plated in a 96-well plate for 24 hrs. Cells were incubated in triplicates in the presence of medium containing ST/nicotine or 0.02% of DMSO which served as a vehicle control in a final volume of 100 µl for 24–96 hrs at 37 °C. Cell death was measured by adding 3-(4,5-dimethylthiazol-2-yl)-2,5-diphenyltetrazolium bromide (MTT) at 37 °C for 3–4 h. The formazan crystals were dissolved in 100 µl of dimethylsulphoxide (DMSO) and optical density (OD) was measured at wavelength of 570 nm. The percentage cell death was calculated individually for each dose as follows: (OD_control_−OD_treated_/OD_control_)×100, as described earlier [49].

### Western blotting and Co-immunoprecipitation (Co-IP) assay

Co-immunoprecipitation (Co-IP) assays and WB were carried out as described by us previously [39]. Whole-cell lysates were prepared from GS (50 µM) or nicotine (10 µM) treated SCC4 cells and protein concentration was determined using the Bradford reagent (Sigma) and equal amounts of proteins (50 µg/lane) were resolved on 12% sodium dodecyl sulfate (SDS)-polyacrylamide gel. The proteins were then electro-transferred onto polyvinylidenedifluoride (PVDF) membrane. After blocking with 5% non-fat milk in Tris-buffered saline (TBS, 0.1 M, pH = 7.4), blots were incubated with specific antibodies as per manufacturer's recommended protocol at 4 °C overnight. Protein abundance of α-tubulin (Santa Cruz Biotechnology, CA) served as a control for protein loading in each lane. Membranes were incubated with HRP-conjugated secondary antibodies, (DAKO Cytomation, Glostrup, Denmark), diluted at an appropriate dilution in 1% BSA, for 2 h at room temperature. After each step, blots were washed three times with Tween (0.1%)-Tris-buffer saline (TTBS). Protein bands were detected by the enhanced chemiluminescence method (ECL, Santa Cruz Biotechnology, CA) on XO-MAT film. Co-IP assays were carried out as described earlier.

### Sub-cellular fractionation: Isolation of cytoplasm and mitochondria

Head and neck cancer cells (2×10^7^) were treated, harvested and washed once with cold 1X PBS and resuspended in isotonic mitochondrial buffer (210 mM mannitol, 70 mM sucrose, 1 mM EGTA, 10 mM Hepes, pH 7.5, 0.1% BSA) containing protease inhibitor cocktail. The resuspended cells were homogenized with a polytron homogenizer operating for four bursts of 10 s each at a setting of 5 and then centrifuged at 2000 g for 3 min to pellet the nuclei and unbroken cells. The supernatant was centrifuged at 13,000 g for 10 min to pellet mitochondria as described [50]. The supernatant was further centrifuged at 15,000 g to pellet light membranes. The resulting supernatant contained the cytosolic fractions. The mitochondria was washed with mitochondrial buffer twice, resuspended with 1% Nonidet P-40 lysis buffer, rocked for 60 min, and then centrifuged at 13,000 g for 10 min at 4 °C. The supernatant containing mitochondrial proteins was collected. Protein (100 µg) from each fraction was subjected to 12% SDS-PAGE and analyzed by western blot analysis using anti-cytochrome c antibody. The purity of the fractions was confirmed by assessing localization of fraction-specific proteins including succinate dehydrogenase for mitochondria.

### Co-localization studies of Akt and Bax in oral cancer cells using confocal laser scan microscopy

Head and neck cancer cells, SCC4 and HSC2, were grown on coverslips in DMEM medium supplemented with 10% FBS at 37 °C and processed for confocal laser scan microscopy as described by us previously [51]. Cells were rinsed in Dulbecco's PBS (DPBS), fixed in methanol for 5 min at −20 °C and incubated with specific primary antibodies, mouse monoclonal anti-Bax and rabbit monoclonal anti-Akt antibody, incubated as a cocktail at 4 °C overnight. After rinsing in phosphate buffer saline- 0.1% Tween (PBST, 1X) the coverslips were incubated with anti-mouse FITC conjugated/anti-rabbit Alexa flour®594 conjugated secondary antibody for 45 min at 37 °C in the dark. Coverslips were washed and counterstained with DAPI for 30 sec. A mouse antibody against human Bax, a rabbit antibody against human Akt, and fluorescein isothiocyanate-conjugated anti-mouse (green) or rhodamine-conjugated anti-rabbit (red) secondary antibodies were used so that cells could be stained simultaneously without cross-reaction. In negative controls, the primary antibodies were replaced by non-immune mouse IgG of the same isotype to ensure specificity (data not shown). Thereafter, the slides were rinsed and mounted in fluorescence mounting medium and examined with Lieca TCS SP2 confocal laser scanning microscopy (CLSM).

## References

[1] Jemal A, Siegel R, Ward E, Hao Y, Xu J (2009). Cancer statistics, 2009.. CA Cancer J Clin.

[2] Parkin DM, Bray F, Ferlay J, Pisani P (2005). Global cancer statistics, 2002.. CA Cancer J Clin.

[3] Sapkota AR, Berger S, Vogel TM (2010). Human pathogens abundant in the bacterial metagenome of cigarettes.. Environ Health Perspect.

[4] McClave AK, Whitney N, Thorne SL, Mariolis P, Dube SR (2010). Adult tobacco survey - 19 States, 2003-2007.. MMWR Surveill Summ.

[5] Colilla SA (2010). An epidemiologic review of smokeless tobacco health effects and harm reduction potential.. Regul Toxicol Pharmacol.

[6] Warnakulasuriya KA, Ralhan R (2007). Clinical, pathological, cellular and molecular lesions caused by oral smokeless tobacco--a review.. J Oral Pathol Med.

[7] Cogliano V, Straif K, Baan R, Grosse Y, Secretan B (2004). Smokeless tobacco and tobacco-related nitrosamines.. Lancet Oncol.

[8] Stepanov I, Villalta PW, Knezevich A, Jensen J, Hatsukami D (2010). Analysis of 23 polycyclic aromatic hydrocarbons in smokeless tobacco by gas chromatography-mass spectrometry.. Chem Res Toxicol.

[9] Dasgupta P, Rizwani W, Pillai S, Kinkade R, Kovacs M (2009). Nicotine induces cell proliferation, invasion and epithelial-mesenchymal transition in a variety of human cancer cell lines.. Int J Cancer.

[10] Davis R, Rizwani W, Banerjee S, Kovacs M, Haura E (2009). Nicotine promotes tumor growth and metastasis in mouse models of lung cancer.. PLoS One.

[11] Nishioka T, Guo J, Yamamoto D, Chen L, Huppi P (2010). Nicotine, through upregulating pro-survival signaling, cooperates with NNK to promote transformation.. J Cell Biochem.

[12] Mai H, May WS, Gao F, Jin Z, Deng X (2003). A functional role for nicotine in Bcl2 phosphorylation and suppression of apoptosis.. J Biol Chem.

[13] West KA, Brognard J, Clark AS, Linnoila IR, Yang X (2003). Rapid Akt activation by nicotine and a tobacco carcinogen modulates the phenotype of normal human airway epithelial cells.. J Clin Invest.

[14] Kurinna SM, Tsao CC, Nica AF, Jiffar T, Ruvolo PP (2004). Ceramide promotes apoptosis in lung cancer-derived A549 cells by a mechanism involving c-Jun NH2-terminal kinase.. Cancer Res.

[15] Zhang J, Kamdar O, Le W, Rosen GD, Upadhyay D (2009). Nicotine induces resistance to chemotherapy by modulating mitochondrial signaling in lung cancer.. Am J Respir Cell Mol Biol.

[16] Urizar NL, Liverman AB, Dodds DT, Silva FV, Ordentlich P (2002). A natural product that lowers cholesterol as an antagonist ligand for FXR.. Science.

[17] Urizar NL, Moore DD (2003). GUGULIPID: a natural cholesterol-lowering agent.. Annu Rev Nutr;.

[18] Meselhy MR (2003). Inhibition of LPS-induced NO production by the oleogum resin of Commiphora wightii and its constituents.. Phytochemistry.

[19] Shishodia S, Aggarwal BB (2004). Guggulsterone inhibits NF-kappaB and IkappaBalpha kinase activation, suppresses expression of anti-apoptotic gene products, and enhances apoptosis.. J Biol Chem.

[20] Samudio I, Konopleva M, Safe S, McQueen T, Andreeff M (2005). Guggulsterones induce apoptosis and differentiation in acute myeloid leukemia: identification of isomer-specific antileukemic activities of the pregnadienedione structure.. Mol Cancer Ther.

[21] Singh SV, Zeng Y, Xiao D, Vogel VG, Nelson JB (2005). Caspase-dependent apoptosis induction by guggulsterone, a constituent of Ayurvedic medicinal plant Commiphora mukul, in PC-3 human prostate cancer cells is mediated by Bax and Bak.. Mol Cancer Ther.

[22] Singh SV, Choi S, Zeng Y, Hahm ER, Xiao D (2007). Guggulsterone-induced apoptosis in human prostate cancer cells is caused by reactive oxygen intermediate dependent activation of c-Jun NH2-terminal kinase.. Cancer Res.

[23] Shishodia S, Sethi G, Ahn KS, Aggarwal BB (2007). Guggulsterone inhibits tumor cell proliferation, induces S-phase arrest, and promotes apoptosis through activation of c-Jun N-terminal kinase, suppression of Akt pathway, and downregulation of antiapoptotic gene products.. Biochem Pharmacol.

[24] An MJ, Cheon JH, Kim SW, Kim ES, Kim TI (2009). Guggulsterone induces apoptosis in colon cancer cells and inhibits tumor growth in murine colorectal cancer xenografts.. Cancer Lett.

[25] Shishodia S, Harikumar KB, Dass S, Ramawat KG, Aggarwal BB (2008). The guggul for chronic diseases: ancient medicine, modern targets.. Anticancer Res.

[26] Sarfaraz S, Siddiqui IA, Syed DN, Afaq F, Mukhtar H (2008). Guggulsterone modulates MAPK and NF-kappaB pathways and inhibits skin tumorigenesis in SENCAR mice.. Carcinogenesis.

[27] Arora S, Matta A, Shukla NK, Deo SV, Ralhan R (2005). Identification of differentially expressed genes in oral squamous cell carcinoma.. Mol Carcinog.

[28] Jayasurya R, Sathyan KM, Lakshminarayanan K, Abraham T, Nalinakumari KR (2005). Phenotypic alterations in Rb pathway have more prognostic influence than p53 pathway proteins in oral carcinoma.. Mod Pathol.

[29] Mishra R, Das BR (2005). Activation of STAT 5-cyclin D1 pathway in chewing tobacco mediated oral squamous cell carcinoma.. Mol Biol Rep.

[30] Soni S, Kaur J, Kumar A, Chakravarti N, Mathur M (2005). Alterations of rb pathway components are frequent events in patients with oral epithelial dysplasia and predict clinical outcome in patients with squamous cell carcinoma.. Oncology.

[31] Kar P, Supakar PC (2006). Expression of Stat5A in tobacco chewing-mediated oral squamous cell carcinoma.. Cancer Lett;.

[32] Kihara T, Shimohama S, Sawada H, Honda K, Nakamizo T (2001). alpha 7 nicotinic receptor transduces signals to phosphatidylinositol 3-kinase to block A beta-amyloid-induced neurotoxicity.. J Biol Chem;.

[33] West R, McNeill A, Raw M (2004). Smokeless tobacco cessation guidelines for health professionals in England.. Br Dent J.

[34] Ho R, Minturn JE, Hishiki T, Zhao H, Wang Q (2005). Proliferation of human neuroblastomas mediated by the epidermal growth factor receptor.. Cancer Res.

[35] Morrison DK (2009). The 14-3-3 proteins: integrators of diverse signaling cues that impact cell fate and cancer development.. Trends Cell Biol.

[36] Tzivion G, Gupta VS, Kaplun L, Balan V (2006). 14-3-3 proteins as potential oncogenes.. Semin Cancer Biol.

[37] Kaur J, Sawhney M, Dattagupta S, Shukla NK, Srivastava A (2010). Clinical significance of Phosphatidyl Inositol Synthase overexpression in oral cancer.. BMC Cancer.

[38] Yamada T, Osawa S, Hamaya Y, Furuta T, Hishida A (2010). Guggulsterone suppresses bile acid-induced and constitutive caudal-related homeobox 2 expression in gut-derived adenocarcinoma cells.. Anticancer Res.

[39] Xu HB, Li L, Liu GQ (2009). Reversal of P-glycoprotein-mediated multidrug resistance by guggulsterone in doxorubicin-resistant human myelogenous leukemia (K562/DOX) cells.. Pharmazie.

[40] Leeman-Neill RJ, Wheeler SE, Singh SV, Thomas SM, Seethala RR (2009). Guggulsterone enhances head and neck cancer therapies via inhibition of signal transducer and activator of transcription-3.. Carcinogenesis.

[41] Kim ES, Hong SY, Lee HK, Kim SW, An MJ (2008). Guggulsterone inhibits angiogenesis by blocking STAT3 and VEGF expression in colon cancer cells.. Oncol Rep.

[42] Sarfaraz S, Siddiqui IA, Syed DN, Afaq F, Mukhtar H (2008). Guggulsterone modulates MAPK and NF-kappaB pathways and inhibits skin tumorigenesis in SENCAR mice.. Carcinogenesis.

[43] De Gottardi A, Dumonceau JM, Bruttin F, Vonlaufen A, Morard I (2006). Expression of the bile acid receptor FXR in Barrett's esophagus and enhancement of apoptosis by guggulsterone in vitro.. Mol Cancer.

[44] Sakai E, Tsuchida N (1992). Most human squamous cell carcinomas in the oral cavity contain mutated p53 tumor-suppressor genes.. Oncogene.

[45] Kim MS, Li SL, Bertolami CN, Cherrick HM, Park NH (1993). State of p53, Rb and DCC tumor suppressor genes in human oral cancer cell lines.. Anticancer Res.

[46] Murugan AK, Hong NT, Fukui Y, Munirajan AK, Tsuchida N (2008). Oncogenic mutations of the PIK3CA gene in head and neck squamous cell carcinomas.. Int J Oncol.

[47] Macha MA, Matta A, Sriram U, Thakkar A, Shukla NK (2009). Clinical significance of TC21 overexpression in oral cancer.. J Oral Pathol Med (In print).

[48] Rohatgi N, Kaur J, Srivastava A, Ralhan R (2005). Smokeless tobacco (khaini) extracts modulate gene expression in epithelial cell culture from an oral hyperplasia.. Oral Oncol.

[49] Sharma C, Kaur J, Shishodia S, Aggarwal BB, Ralhan R (2006). Curcumin down regulates smokeless tobacco-induced NF-kappaB activation and COX-2 expression in human oral premalignant and cancer cells.. Toxicology.

[50] Kuhar M, Sen S, Singh N (2006). Role of mitochondria in quercetin-enhanced chemotherapeutic response in human non-small cell lung carcinoma H-520 cells.. Anticancer Res.

[51] Sawhney M, Matta A, Macha MA, Kaur J, DattaGupta S (2009). Cytoplasmic accumulation of activated leukocyte cell adhesion molecule is a predictor of disease progression and reduced survival in oral cancer patients.. Int J Cancer.

